# Polymer Network-Confined Purely Organic Material with Long-Lived Delayed Emission for Aqueous Iron(III) Ion Sensing

**DOI:** 10.3390/molecules31152575

**Published:** 2026-07-24

**Authors:** Rao Luo, Xiaohan Lin, Chen Xu, Shaodong Zhou, Chao Qian

**Affiliations:** 1 Polytechnic Institute of Zhejiang University, Zhejiang University, Hangzhou 310015, China; 12560064@zju.edu.cn (R.L.); szhou@zju.edu.cn (S.Z.); 2 Institute of Zhejiang University—Quzhou, Quzhou 324000, China

**Keywords:** thermally activated delayed fluorescence, polymer network confinement, transmembrane diffusion model

## Abstract

Luminescent sensing in aqueous media using organic small-molecule emitters is often constrained by water-induced fluorescence quenching and indicator leakage. In this study, a polymer-confined thermally activated delayed fluorescence (TADF) material, poly-BrTPPz, was synthesized by covalently copolymerizing a donor–acceptor monomer into a polyacrylamide network. Density functional theory calculations indicate spatial frontier orbital separation. The polymer matrix restricts intramolecular motion, while the polar amide microenvironment provides a solid-state solvation effect, decreasing the reverse intersystem crossing barrier to activate delayed luminescence with a lifetime of 303 μs and a photoluminescence quantum yield of 69.1% in the solid state. In aqueous environments, the material exhibits a selective quenching response toward iron(III) ions (Fe^3+^) through a mechanism involving the inner filter effect and pyrazine-coordinated static quenching. To mitigate potential secondary environmental contamination, a transmembrane diffusion model was evaluated by encapsulating the polymer within a semi-permeable membrane, which limits indicator leakage while permitting analyte permeation. This work outlines a design approach for environment-responsive luminescent devices in closed aquatic systems.

## 1. Introduction

Optical and fluorescence sensing technologies have emerged as robust tools for environmental monitoring and biological analysis due to their operational simplicity and non-destructive nature [1,2,3,4,5,6]. In aquatic chemistry, traditional responsive materials often target metal contaminants such as ferric ions (Fe^3+^) [7,8]. Various fluorescent probes based on carbon dots [9,10,11], metal–organic frameworks (MOFs) [12,13,14], and small organic molecules [15,16,17] have been reported, providing diverse strategies for Fe^3+^ identification. However, conventional organic systems based on nanosecond fluorescence are limited by their short emission lifetimes, rendering them highly susceptible to severe background autofluorescence and scattering interference in complex matrices [18,19,20,21].

Purely organic thermally activated delayed fluorescence (TADF) materials, characterized by a small singlet-triplet energy gap (∆EST), can effectively harness triplet excitons via the reverse intersystem crossing (RISC) process. This results in micro-to-millisecond delayed emission, offering immense potential for high-contrast time-resolved sensing [22,23,24,25,26,27]. Nevertheless, the practical deployment of free TADF small molecules in water remains hindered by severe aqueous quenching via high-frequency polar network vibrations [28,29], alongside the prominent risks of dye leakage and secondary pollution [30,31,32].

**Scheme 1:**
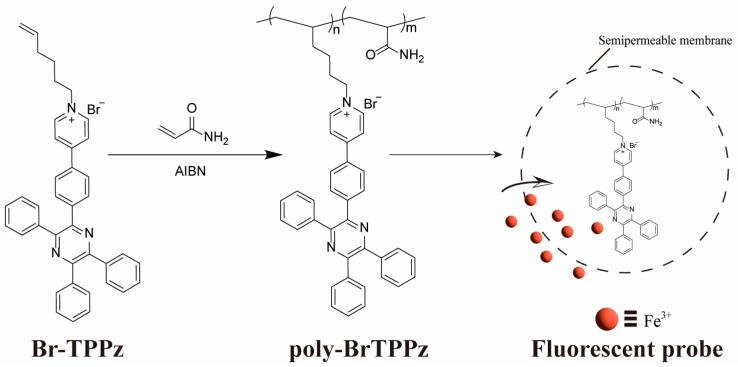
Synthetic route of the polymer and transmembrane design for Fe^3+^.

To overcome these bottlenecks, incorporating TADF luminophores into polymer matrices represents a promising structural confinement strategy (Scheme 1). Herein, we report a polymer-confined TADF material, poly-BrTPPz, based on a sterically hindered donor–acceptor (D-A) monomer Br-TPPz whose frontier molecular orbital separation was verified by DFT calculations [33,34,35]. To mitigate non-radiative losses in water, the TADF luminophore was covalently copolymerized into a polyacrylamide (PAM) network [36,37]. Benefiting from the matrix rigidity endowed by the dense hydrogen-bonding network that restricts intramolecular motion (RIM), together with the solid-state solvation effect of the polar amide microenvironment, the RISC energy barrier is significantly minimized to activate efficient delayed luminescence [38,39,40]. To evaluate its potential for environmental sensing, the optical response of poly-BrTPPz to various aqueous metal ions was investigated. The polymer exhibited highly selective fluorescence quenching by Fe^3+^, driven by both the inner filter effect (IFE) and ground-state static quenching [41,42]. Furthermore, encapsulating poly-BrTPPz within a semi-permeable membrane established a transmembrane sensing model (Scheme 1) that eliminates probe leakage while enabling specific analyte permeation. Overall, this work elucidates the photophysics of polymer-confined excitons in aqueous media and provides a design paradigm for zero-pollution, stimuli-responsive luminescent sensors in closed systems.

## 2. Results

### 2.1. Theoretical Calculations

**Figure 1:**
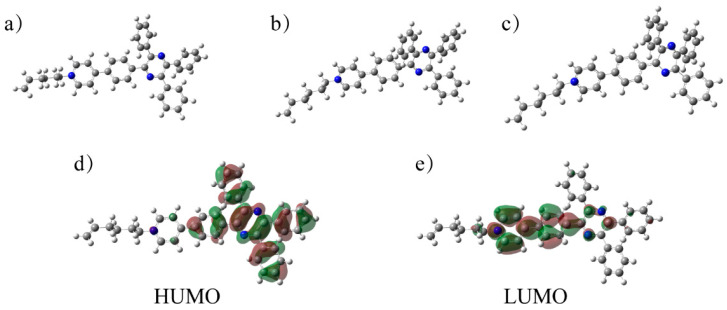
Optimized geometries of the monomer Br-TPPz in the (**a**) S_0_, (**b**) S_1_, and (**c**) T_1_ states in an aqueous solution. Spatial distributions of the (**d**) HOMO and (**e**) LUMO of Br-TPPz in an aqueous solution.

Density functional theory (DFT) calculations were performed to systematically investigate the ground- and excited-state electronic structures of the monomer in an aqueous environment. The optimized ground-state (S_0_) geometry (Figure 1a) exhibits a highly twisted dihedral angle between the sterically hindered triphenylpyrazine donor and the central phenyl acceptor. This non-coplanar conformation effectively disrupts the delocalized conjugation along the molecular backbone. The frontier molecular orbital (FMO) distributions (Figure 1d,e) further corroborate its strong intramolecular charge transfer (ICT) characteristic: the highest occupied molecular orbital (HOMO) is predominantly localized on the electron-rich triphenylpyrazine donor, whereas the lowest unoccupied molecular orbital (LUMO) is concentrated on the electron-deficient phenyl-pyridinium acceptor. Such distinct spatial orbital separation effectively minimizes the electron exchange energy.

Time-dependent DFT (TD-DFT) calculations reveal that the theoretical ∆EST of the free monomer is approximately 0.3 eV. In photophysical dynamics, a barrier of 0.3 eV remains relatively high compared to room-temperature thermal energy, indicating a thermodynamic hurdle for efficient RISC to occur directly in the solvated free monomer. Consequently, excellent TADF performance is postulated to be difficult to achieve in the free monomer alone. However, by covalently copolymerizing the luminophore into a PAM network, the highly polar microenvironment and the rigid spatial restriction imposed by the dense hydrogen-bonding network can exert a synergistic confinement effect. This effect is expected to further narrow the operational energy gap, thereby activating the TADF emission in aqueous media. Furthermore, geometry optimizations of the S_1_ and T_1_ excited states (Figure 1b,c) suggest that the molecule undergoes structural relaxation upon photoexcitation to stabilize the highly polar charge transfer (CT) state. Notably, the molecular geometries of the S_1_ and T_1_ states display a high degree of consistency. This conformational similarity in the excited states is highly beneficial for reducing the non-adiabatic coupling barrier, thereby facilitating efficient state-to-state crossing.

### 2.2. Photophysical Properties

**Figure 2:**
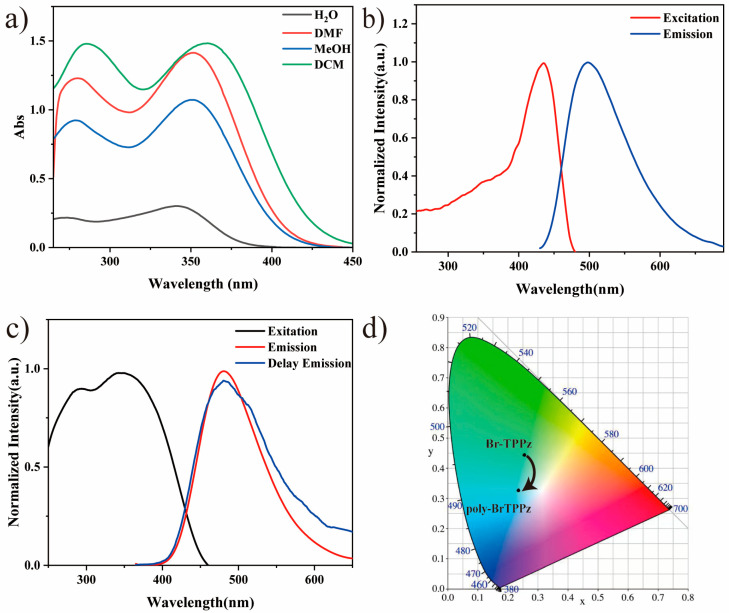
(**a**) UV-vis absorption spectra of monomer Br-TPPz in various solvents. (**b**) Photoluminescence excitation and emission spectra of Br-TPPz in the solid state. (**c**) Excitation, steady-state emission, and delayed emission spectra of poly-BrTPPz in the solid state. (**d**) CIE 1931 chromaticity diagram.

To investigate the ground-state electronic transition characteristics, UV-vis absorption spectra of the monomer Br-TPPz were recorded in various solvents with differing polarities (Figure 2a). The spectra exhibit typical dual-absorption features: the high-energy bands (280–290 nm) are primarily assigned to the local p-p* transitions of the triphenylpyrazine and phenyl moieties, while the broad, featureless absorption band in the low-energy region (350–360 nm) originates from the ICT transition from the electron-rich triphenylpyrazine donor to the strongly electron-deficient pyridinium acceptor. Notably, the ICT absorption peak position remains nearly stationary despite the drastic increase in solvent polarity from DCM to H_2_O. This negligible solvatochromic behavior in the ground state suggests that the pyridinium cation endows the monomer with a pre-existing, robust dipole moment, rendering its ground-state electronic distribution less sensitive to the surrounding solvent environment.

The excited-state relaxation dynamics were further elucidated through steady-state PL spectroscopy of the monomer in the solid state (Figure 2b). The excitation spectrum (red line, peak at 433 nm) matches the low-energy tail of the absorption spectrum, confirming that the luminescence originates from the low-lying ICT transition. Upon excitation at 400 nm, the monomer exhibits a broad, featureless emission centered at approximately 500 nm, corresponding to the blue-green region. A significant Stokes shift of 67 nm was observed, characteristic of a strong ICT excited state. This large Stokes shift, coupled with the broad emission profile, indicates that the molecule undergoes substantial geometric relaxation from the Franck-Condon state to the lowest excited-state minimum due to the pronounced charge separation and steric hindrance between the D-A units [43,44]. This observation aligns with the UV-vis findings, further substantiating the prominent ICT nature of the cationic monomer.

The photophysical properties of the polymer were investigated using steady-state and time-resolved delayed emission spectroscopy. As shown in Figure 2c, poly-BrTPPz displays a broad excitation band with a peak at 355 nm, consistent with the monomer’s absorption, indicating that the electronic structure of the luminophore remains intact after polymerization. Upon 355 nm excitation, the polymer exhibits steady-state emission at 480 nm (Figure 2c, red line). Remarkably, compared to the monomer’s emission (500 nm), poly-BrTPPz shows a significant blue shift, manifesting as a transition from green to blue emission in the CIE 1931 chromaticity diagram (Figure 2d). This blue shift is attributed to the rigid microenvironment provided by the dense hydrogen-bonding network within the PAM matrix. The rigid microenvironment of the polymer matrix severely restricts the intramolecular motions and excited-state conformational twisting of the emissive molecules, efficiently cutting off the non-radiative energy dissipation networks [45]. Therefore, the excitons are trapped in a local potential minimum at a higher energy level on the potential energy surface. The lack of sufficient conformational relaxation thereby drives the radiative recombination directly from the less-relaxed, high-energy state. Simultaneously, the non-conjugated PAM chains provide spatial isolation, minimizing non-radiative losses induced by aggregation. The delayed emission spectrum (Figure 2c, blue line) closely resembles the steady-state profile in both peak position and shape, suggesting that both short-lived and long-lived emissions originate from the same singlet charge transfer (^1^CT) state.

**Figure 3:**
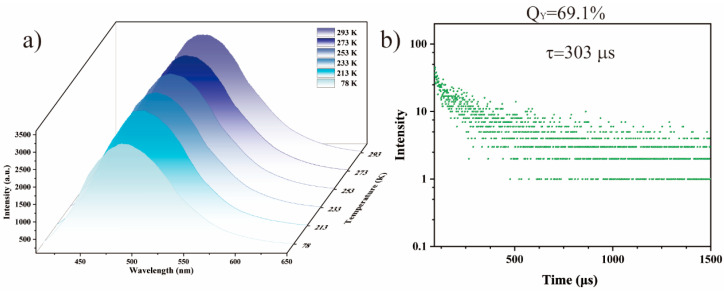
(**a**) Temperature-dependent photoluminescence spectra of solid poly-BrTPPz. (**b**) Time-resolved transient photoluminescence decay curve of solid poly-BrTPPz at room temperature.

To deeply elucidate and verify the mechanism of this long-lived luminescence, the temperature-dependent photoluminescence spectra of the polymer solid were systematically investigated (Figure 3a). In conventional conjugated luminescent polymers, elevated temperatures typically intensify the internal thermal vibrations of the polymer chains. This increased thermal energy activates various non-radiative relaxation channels (e.g., exciton-phonon coupling), leading to severe thermal quenching [46,47]. In stark contrast, the temperature-dependent spectra of polymer systems exhibit a distinct kinetic evolution characteristic of TADF. As shown in Figure 3a, upon heating from 78 K to 293 K, the emission displays two notable features: an anomalous intensity enhancement and a regular redshift of the emission peak. At the cryogenic temperature of 78 K, the RISC process from the T_1_ to the S_1_ is thermodynamically restricted. Consequently, the spectrum is dominated by the short-lived prompt fluorescence resulting from direct exciton relaxation, peaking at 482 nm. As the temperature climbs to 293 K, the macroscopic emission intensity increases drastically, and the peak red-shifts to 498 nm, demonstrating pronounced TADF behavior. Based on these spectroscopic behaviors, the PAM backbone is postulated to impose significant steric hindrance on the pendant D-A luminophores, thereby modulating the overlap of frontier orbitals. Furthermore, the dense amide groups within the polymer network construct a solid-state microenvironment with high local polarity. Through dipole–dipole interactions, this polar environment stabilizes the highly dipolar ^1^CT state, driving the S1 energy level downward. Combining the spectroscopic evidence with the matrix characteristics, the synergistic effect of the polar microenvironment and the rigid backbone effectively minimizes the transition energy barrier, facilitating efficient RISC at room temperature.

To exclude nanosecond prompt fluorescence interference, time-resolved transient decay spectra of the polymer solid at room temperature were recorded using a time-gated technique (Figure 3b). The delayed emission follows a single-exponential decay profile in a semi-logarithmic plot, yielding a fitted delayed lifetime of 303 μs and a photoluminescence quantum yield (PLQY) of 69.1%. Given the nearly identical profiles of the steady-state and delayed emission spectra discussed earlier, this long-lived component is unambiguously assigned to the TADF emission originating from the thermally activated upconversion of T1 excitons to S1 via RISC. Notably, this microsecond-scale long-lived luminescence significantly broadens the dynamic interaction time window between the emitting excitons and target quenchers. This unique attribute lays an ideal photophysical foundation for the subsequent realization of high-sensitivity, time-resolved sensing that is intrinsically immune to spontaneous background autofluorescence interference.

### 2.3. Stimuli-Responsive Properties and Application in Closed-System Sensing

**Figure 4:**
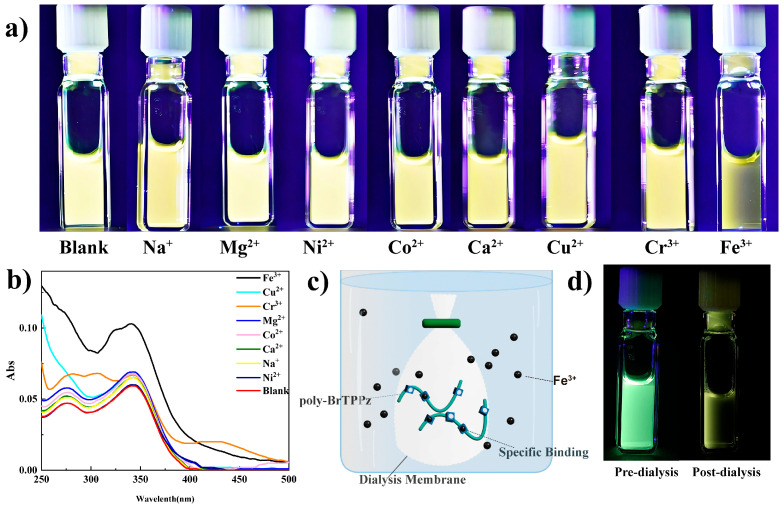
(**a**) Photographs of poly-BrTPPz aqueous solutions in the presence of various metal ions. (**b**) UV-vis absorption spectra of poly-BrTPPz upon the addition of different metal cations. (**c**,**d**) Fluorescence photographs of the solution before and after dialysis.

To unravel the highly specific quenching mechanism of poly-BrTPPz toward Fe^3+^, the UV-vis absorption spectra of the polymer solutions in the absence and presence of various metal ions were systematically recorded (Figure 4b). Upon the introduction of the target analyte, Fe^3+^, a broad and intense absorption band emerged in the range of 250–450 nm, thoroughly obscuring the intrinsic absorption peak of the polymer at 340 nm. This phenomenon indicates severe competitive absorption, signifying that the inner filter effect (IFE) acts as a primary contributor to the observed fluorescence quenching.

The responsiveness toward Fe^3+^ can be rationalized at the molecular level by correlating the coordination chemistry of the copolymerized luminophore with the electronic structures evaluated in Section 2.1. Hydrophilically, while the PAM matrix provides an aqueous diffusion pathway, the binding behavior occurs at the electron-rich pyrazine units of the embedded Br-TPPz monomers. The pyrazine moiety, bearing lone-pair electrons, serves as a complexing site for target cations within the matrix [48]. This electronic susceptibility corresponds to the frontier molecular orbital characteristics established in Section 3.1. Because the separated HOMO-LUMO distribution of Br-TPPz governs its intrinsic ICT state, the LUMO is localized on the pyrazine-based electron acceptor (Figure 1e). Coordination of the Lewis acidic Fe^3+^ to the pyrazine nitrogen alters the electronic potential of the acceptor site due to its electron-withdrawing effect and paramagnetic nature [49]. Consequently, this proximal microperturbation facilitates photoinduced electron transfer (PET) from the donor to the metal-coordinated acceptor or enhances paramagnetic spin-orbital coupling, opening non-radiative decay channels that suppress the ^1^CT state emission [50].

Minimizing the risks of secondary environmental pollution typically associated with traditional small-molecule systems remains a paramount consideration in material selection; hence, a transmembrane diffusion model was deployed to evaluate the feasibility of this macromolecular material for closed-system water monitoring. Operationally, the aqueous solution of **poly-BrTPPz** was encapsulated inside a dry regenerated cellulose dialysis bag (MWCO = 500 Da, JielePu), followed by complete immersion into an external aqueous environment containing Fe^3+^ (Figure 4c). Intense green fluorescence was exhibited by the polymer solution under UV irradiation prior to dialysis, whereas substantial macroscopic quenching was observed for the solution collected after transmembrane permeation (Figure 4d). Substantial macroscopic quenching was subsequently observed within the dialysis bag after a designated period of transmembrane permeation. This visual response stems from the unobstructed diffusion of small-sized Fe^3+^ ions across the semi-permeable membrane into the polyacrylamide network, triggering effective physical and optical interactions with the confined luminophores. Concurrently, stable retention of the macromolecular polymer matrix within the membrane boundaries fundamentally precludes the leakage of the luminescent materials into the surrounding aquatic environment. Such a collaborative mechanism of structural retention and selective analyte permeation successfully demonstrates the viability of utilizing polymer-confined TADF materials for pollution-free environmental monitoring in closed systems.

## 3. Materials and Methods

### 3.1. Material

Analytical-grade solvents and chemical reagents were procured from commercial vendors (TCI Chemicals (Tokyo, Japan), Macklin^®^ (Shanghai, China), or Adamas-beta^®^ (Shanghai, China)) and utilized as received without extra purification, except where specifically noted. All aqueous solutions involved in the testing procedures were prepared with ultrapure water. The target macromolecules were obtained via free-radical polymerization methods. Furthermore, structural confirmation for the newly prepared compounds was performed using ^1^H NMR, ^13^C NMR, alongside electrospray ionization liquid chromatography-mass spectrometry (ESI-LC-MS).

### 3.2. Synthesis of Polymers

The target polymer was synthesized via free-radical copolymerization with a feed molar ratio of Br-TPPz monomer to acrylamide of 1:50. Typically, Br-TPPz (40 mg, 0.064 mmol), acrylamide (227 mg, 3.19 mmol), and *N*,*N*-dimethylformamide (DMF, 4 mL) were charged into a 20 mL Schlenk tube. The resulting solution was degassed with argon for 30 min. Subsequently, 2,2′-azobis(2-methylpropionitrile) (AIBN, 6 mg) was added as the radical initiator, and the mixture was stirred at 65 °C for 12 h under an argon atmosphere. After the reaction, methanol (5 mL) was added to the mixture to precipitate the polymeric product. The precipitate was collected and thoroughly washed with methanol (3 × 5 mL) to afford the purified polymer. The synthesized polymer was characterized by aqueous gel permeation chromatography (GPC).

### 3.3. Characterization Methods

^1^H and ^13^C NMR spectra were recorded on a Bruker AVANCE NEO 500 spectrometer (Billerica, MA, USA). ESI-LC-MS spectra were recorded on a SHIMADZU LCMS-IT-TOF system (Kyoto, Japan). UV-vis absorption spectra were measured on a Varian Cary 500 spectrophotometer (Melbourne, Australia). Photoluminescence (PL) emission spectra and time-resolved decay spectra were recorded on an Edinburgh FLS1000 spectrofluorometer (Edinburgh, UK). Temperature-dependent fluorescence spectra were measured on a Horiba iHR550 spectrometer (Kyoto, Japan). Aqueous gel permeation chromatography (GPC) measurements were performed on an Agilent 1260 chromatograph (Santa Clara, CA, USA). The columns used in GPC were a series of Waters Ultrahydrogel (length/i.d. = 300 × 7.8 mm^2^; pore sizes = 500, 250, and 120 Å; the mobile phase was a 0.1 M NaNO_3_ aqueous solution; flow rate = 1.0 mL/min). These characterization results are provided in the Supporting Information.

## 4. Conclusions

In summary, an effective strategy to modulate TADF performance through covalent confinement within a polymer matrix was developed, yielding a purely organic luminescent material, poly-BrTPPz, capable of delayed emission in aqueous media. Although effective frontier orbital separation is achieved by the sterically hindered D-A monomer Br-TPPz, a high transition energy barrier and significant non-radiative dissipation in its free state are indicated by theoretical calculations. Covalent copolymerization of the luminophore into a PAM network was executed to construct a rigid polymer scaffold. RIM is successfully induced by this matrix, thereby suppressing the non-radiative deactivation of triplet excitons. Concurrently, lowering of the transition energy barrier is achieved through the solid-state solvation effect provided by the polar microenvironment of the polymer backbone. Driven by the synergy between matrix restriction and polar stabilization, activation of the RISC process is accomplished, yielding a delayed fluorescence lifetime of 303 ms. Owing to these long-lived exciton dynamics and the coordination affinity of the polymer network, a highly selective quenching response toward Fe^3+^ in water was exhibited by the material as a stimuli-responsive application. Transmembrane permeation evaluations using a dialysis bag model confirmed the retention capability of this macromolecular structure, coupled with a responsive quenching behavior toward external Fe^3+^. Implementation of this collaborative matrix retention and selective analyte permeation highlights the potential of the system for pollution-free environmental monitoring in closed systems. Elucidation of the structure–property relationships in this study outlines a reliable design paradigm for developing novel purely organic aqueous luminescent materials and environment-responsive devices.

## Data Availability

The data supporting this work is in the article and Supplementary Materials.

## Supplementary Materials

The following supporting information can be downloaded at: https://www.mdpi.com/article/10.3390/molecules31152575/s1.
