# Supplementary material for: Polymer Network-Confined Purely Organic Material with Long-Lived Delayed Emission for Aqueous Iron(III) Ion Sensing
Source: Molecules. 2026 Jul 24;31(15):2575. doi: 10.3390/molecules31152575 (PMC13468616; doi:10.3390/molecules31152575)
Supplement: Supplementary file 1 [file molecules-31-02575-s001.zip › molecules-4406361-supplementary.pdf]

Supporting Information

Polymer Network-Confined Purely Organic Material with Long-Lived Delayed Emission for Aqueous Iron(III) Ion Sensing

Rao Luo [1], Xiaohan Lin [1,2,*], Chen Xu [1,2,*], Shaodong Zhou [1,2] and Chao Qian [1,2,*]

[1] Polytechnic Institute of Zhejiang University, Zhejiang University, Hangzhou 310015, China; 12560064@zju.edu.cn (R.L.); szhou@zju.edu.cn (S.Z.)
[2] Institute of Zhejiang University—Quzhou, Quzhou 324000, China
* Correspondence: linxiaohan@zju.edu.cn (X.L.); xuchenn@zju.edu.cn (C.X.); qianchao@zju.edu.cn (C.Q.)

•Contents

Scheme S1. Synthetic routes of target compounds.

Figure S1-S9. ^{1}H NMR spectra, ^{13}C NMR spectra and HRESI-MS spectra of target compounds.

Figure S10. GPC chromatogram plot of poly-BrTPPz.

Table S1. GPC data of poly-BrTPPz.

Table S2. Photophysical properties and temperature-dependent emission of Br-TPPz and poly-Br-TPPz

Table S3. Optimized Cartesian coordinates (in AA) for the ground state (S_0) of Br-TPPz monomer in aqueous solution (M06-2X/def2-TZVP, SMD model).

Materials and Characterization Methods

All reagents were commercially available (Adamas-beta®, Maclin® or TCI Chemicals), all reagents and the solvents of analytical grade were used directly without further purification unless indicated otherwise. Water used in tests was ultrapure. All polymers were synthesized utilizing the free radical polymerization. All the synthesized molecules in the study were characterized by ^{1}H NMR, ^{13}C NMR, and ESI liquid chromatograph-mass spectrometer (ESI-LC-MS). The semi-permeable dialysis membrane (dry regenerated cellulose membrane, MWCO = 500 Da, product model: MD34-500) was purchased from JielePu and handled according to standard protocols before use.

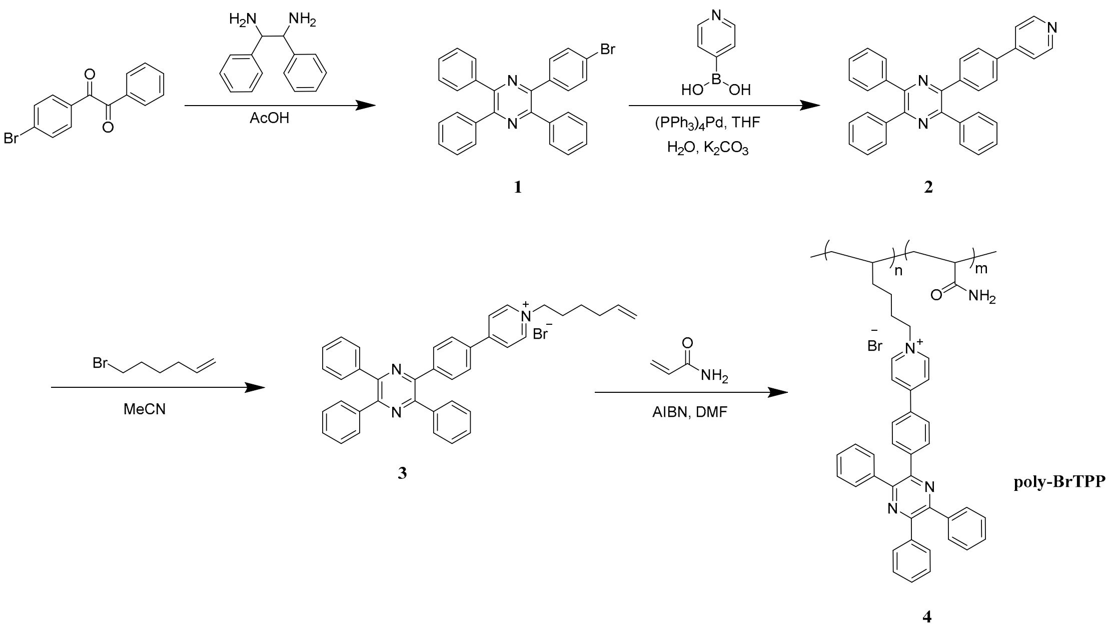

Scheme S1. Synthesis route of target compounds.

Synthesis of 2-(4-bromophenyl)-3,5,6-triphenylpyrazine (1).

1-(4-Bromophenyl)-2-phenylethane-1,2-dione (553 mg, 1.9 mmol) and 1,2-diphenylethane-1,2-diamine (407 mg, 1.9 mmol) were dissolved in AcOH (5 mL) at room temperature. The mixture was then heated to 118 °C and refluxed for 4 h. After cooling to room temperature, the precipitate was collected by vacuum filtration and washed with petroleum ether (PE). The collected crude product was dried in an oven and subsequently stored in a desiccator. Isolated mass: 0.428 g. Yield: 48.6%. ^{1}H NMR (500 MHz, $CDCl_3$) δ 7.58-7.68 (m, 6H), 7.53 (d, J = 8.0 Hz, 2H), 7.45 (d, J = 8.0 Hz, 2H), 7.29-7.39 (m, 9H). ^{13}C NMR (126 MHz, $CDCl_3$) δ 148.75, 148.55, 148.36, 147.14, 138.31, 138.19, 137.42, 131.50, 131.49, 131.48, 129.90, 129.86, 128.86,

128.85, 128.76, 128.46, 128.45, 128.30, 128.29, 123.15. LCMS (m/z): $[M+H]^+$ calcd for $[C_{28}H_{20}BrN_2]^+$, 465.0784; found, 465.0761.

Synthesis of 2,3,5-triphenyl-6-(4-(pyridine-4-yl)phenyl)pyrazine (2). To a two-necked flask, compound **1** (428 mg, 0.92 mmol) and 4-pyridineboronic acid (183 mg, 1.49 mmol) were dissolved in THF (25 mL). An aqueous solution of K_2CO_3 (2 M) was then added. The mixture was degassed and backfilled with argon. Subsequently, $Pd(PPh_3)_4$ was added as the catalyst. The reaction mixture was heated to 68 °C and stirred under reflux for 18 h. Upon completion, the mixture was concentrated under reduced pressure to remove THF. The residue was extracted with EA and saturated brine. The combined organic layers were dried over anhydrous Na_2SO_4, filtered, and concentrated. The crude product was purified by column chromatography to afford compound **2**. Isolated mass: 0.286 g. Yield: 67%. ^{1}H NMR (500 MHz, $CDCl_3$) δ 8.672 (d, J = 6.0 Hz,2H), 7.778 (d, J=8.0 Hz, 2H), 7.57-7.73 (m, 10H), 7.27-7.43 (m, 9H). ^{13}C NMR (126 MHz, $CDCl_3$) δ 150.32, 148.74, 148.58, 148.54, 147.68, 147.42, 139.37, 138.37, 138.34, 138.33, 138.10, 130.66, 129.93, 129.88, 128.85, 128.78, 128.76, 128.44, 128.33, 128.30, 127.44, 126.86, 121.55. LCMS (m/z): $[M+H]^+$ calcd for $[C_{33}H_{24}N_3]^+$, 462.1965; found, 462.1952.

Synthesis of 1-(hex-5-en-1-yl)-4-(4-(3,5,6-triphenylpyrazin-2-yl)phenyl)pyridine-1-ium bromide (3, Br-TPPz). Compound **2** (286 mg, 0.62 mmol) was dissolved in a mixture of DMF (10 mL) and MeCN (12 mL) and heated in an oil bath at 85 °C. 6-Bromo-1-hexene (332 µL, 2.48 mmol) was added via a micropipette. The reaction mixture was maintained at 85 °C for 55 h. After cooling, MeCN was removed by rotary evaporation. The resulting white precipitate was collected by vacuum filtration and dried to afford compound **3** (monomer Br-TPPz). Isolated mass: 0.297 g. Yield: 76.7%. ^{1}H NMR (500 MHz, DMSO-d_6) δ 9.14-9.21 (m, 2H), 8.60 (d, J = 6.5 Hz, 2H), 8.16 (d, J = 8.5 Hz, 2H), 7.84 (d, J = 8.5 Hz, 2H), 7.63-7.67 (m, 2H), 7.58-7.63 (m, 4H), 7.39-7.48 (m, 9H), 5.78-5.89 (m, 1H), 5.04-5.12 (m, 1H), 4.98-5.04 (m, 1H), 4.60-4.68 (m, 2H), 2.09-2.16 (m, 2H), 1.95-2.03 (m, 2H), 1.39-1.48 (m, 2H). ^{13}C NMR (126 MHz, $CDCl_3$) δ 154.70, 149.80, 149.64, 149.43, 147.90, 145.73, 142.56, 139.00, 138.78, 138.75, 138.69, 134.43, 131.71, 130.66, 130.57, 130.53, 130.02, 129.92, 129.89, 129.87, 129.44, 129.32, 129.04, 125.44, 116.27, 60.70, 33.41, 31.12, 25.58. LCMS (m/z): $[M-Br^-]^+$ calcd for $[C_{39}H_{34}N_3]^+$, 544.2747; found, 544.2725.

Preparation of poly-BrTPPz. The target polymer was synthesized via free-radical copolymerization

with a feed molar ratio of Br-TPPz monomer to acrylamide of 1:50. Typically, Br-TPPz (40 mg, 0.064 mmol), acrylamide (227 mg, 3.19 mmol), and *N*,*N*-dimethylformamide (DMF, 4 mL) were charged into a 20 mL Schlenk tube. The resulting solution was degassed with argon for 30 min. Subsequently, 2,2′-azobis(2-methylpropionitrile) (AIBN, 6 mg) was added as the radical initiator, and the mixture was stirred at 65 °C for 12 h under an argon atmosphere. After the reaction, methanol (5 mL) was added to the mixture to precipitate the polymeric product. The precipitate was collected and thoroughly washed with methanol (3×5 mL) to afford the purified polymer. Isolated mass: 0.148 g.

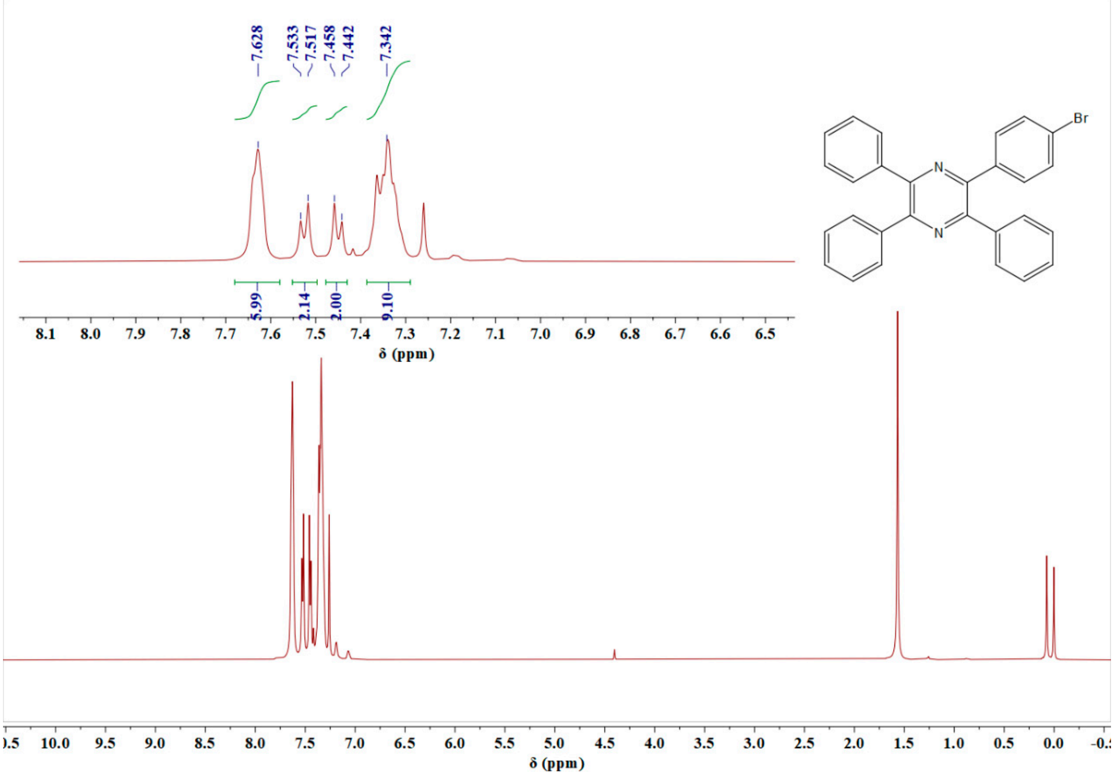

Figure S1. ^{1}H NMR of spectrum of compound **1** in $CDCl_3$.

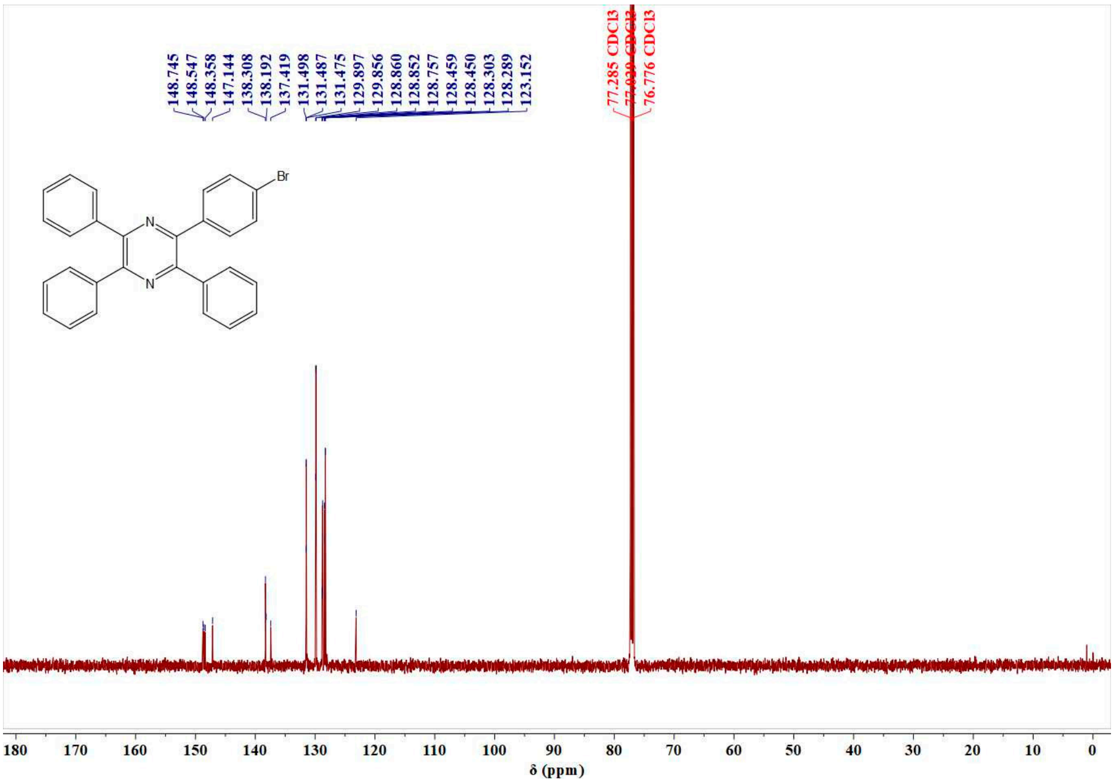

Figure S2. ^{13}C NMR of spectrum of compound **1** in $CDCl_3$.

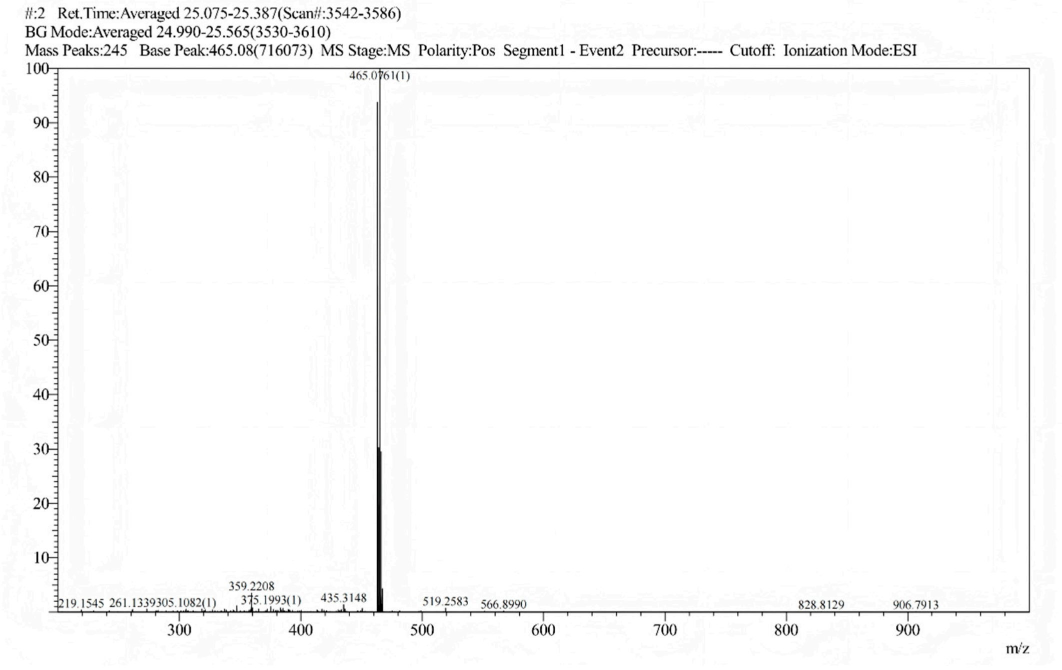

Figure S3. LCESI-MS spectrum of compound **1**.

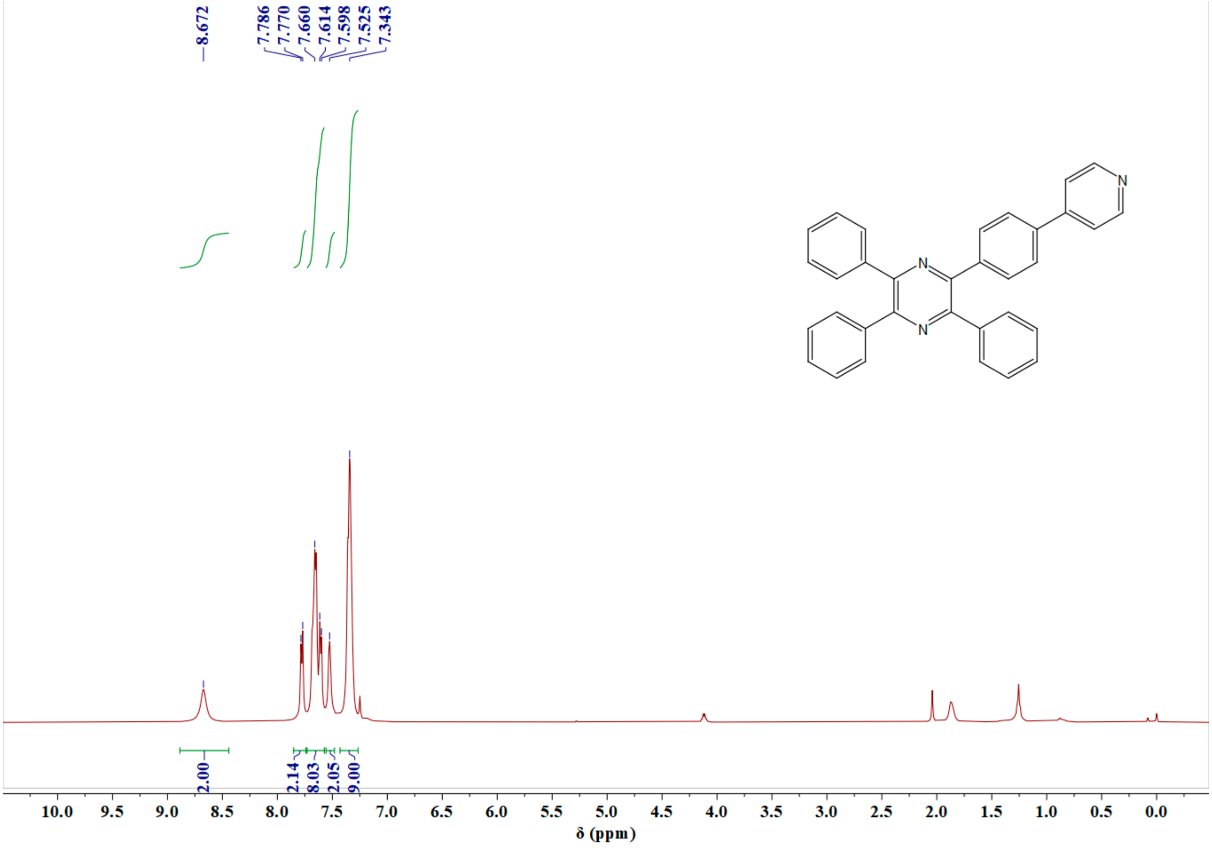

Figure S4. ^{1}H NMR of spectrum of compound **2** in $CDCl_3$.

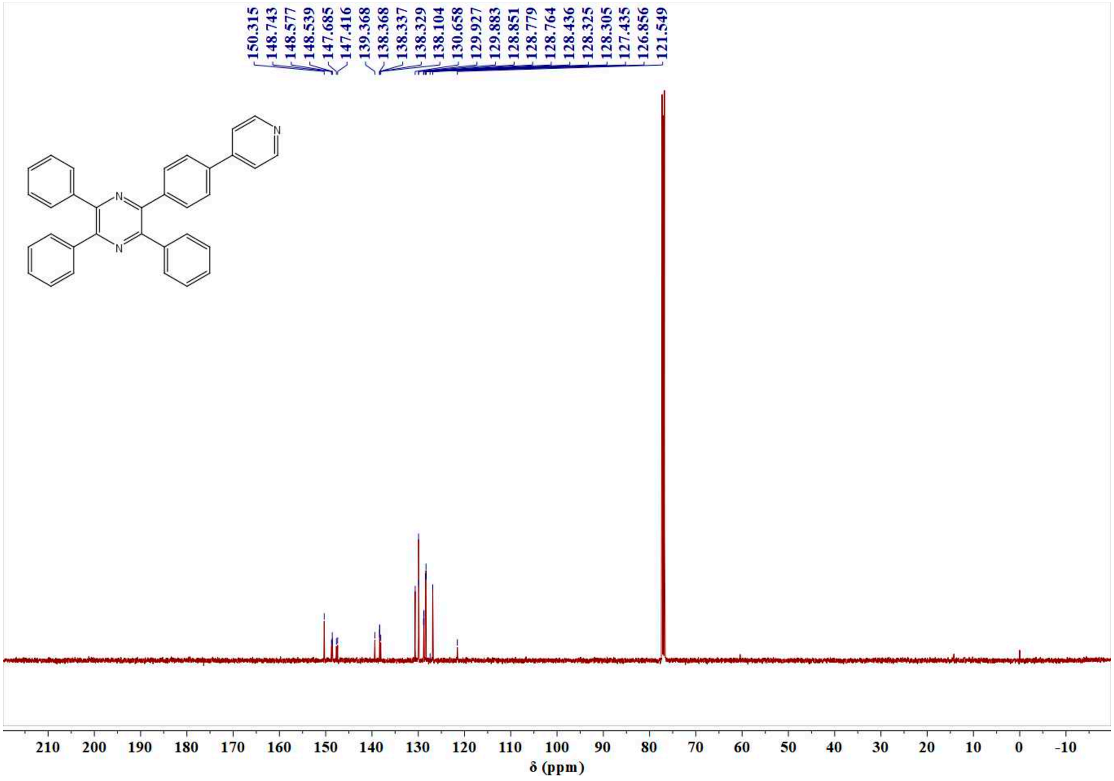

Figure S5. ^{13}C NMR of spectrum of compound **2** in $CDCl_3$.

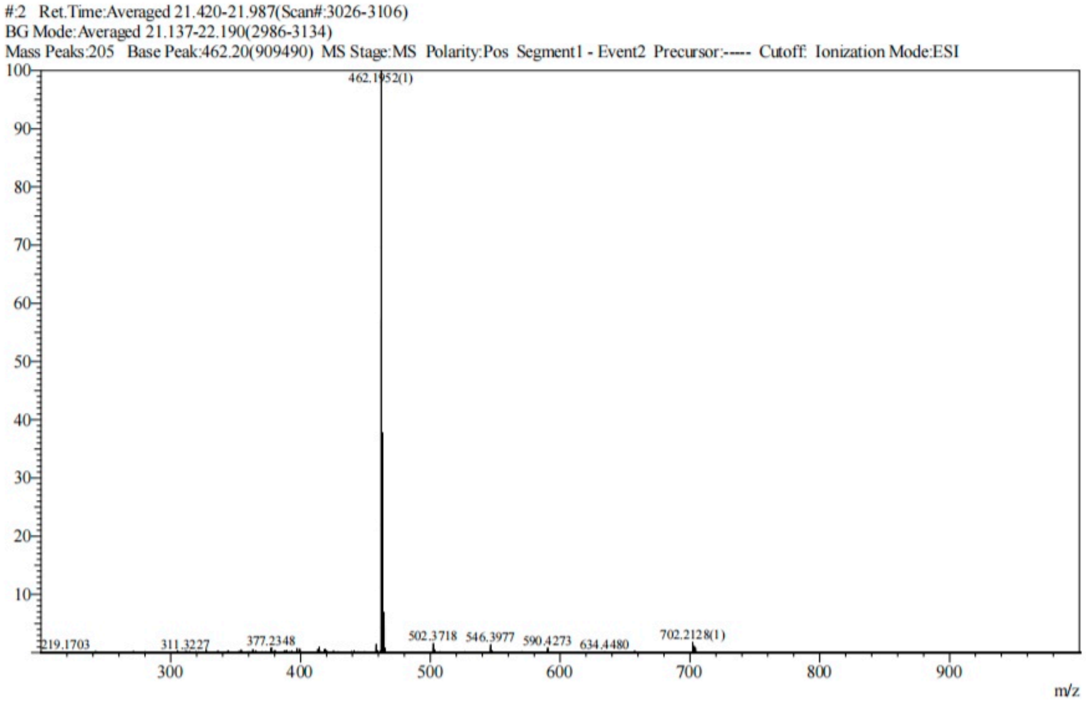

Figure S6. LCESI-MS spectrum of compound **2**.

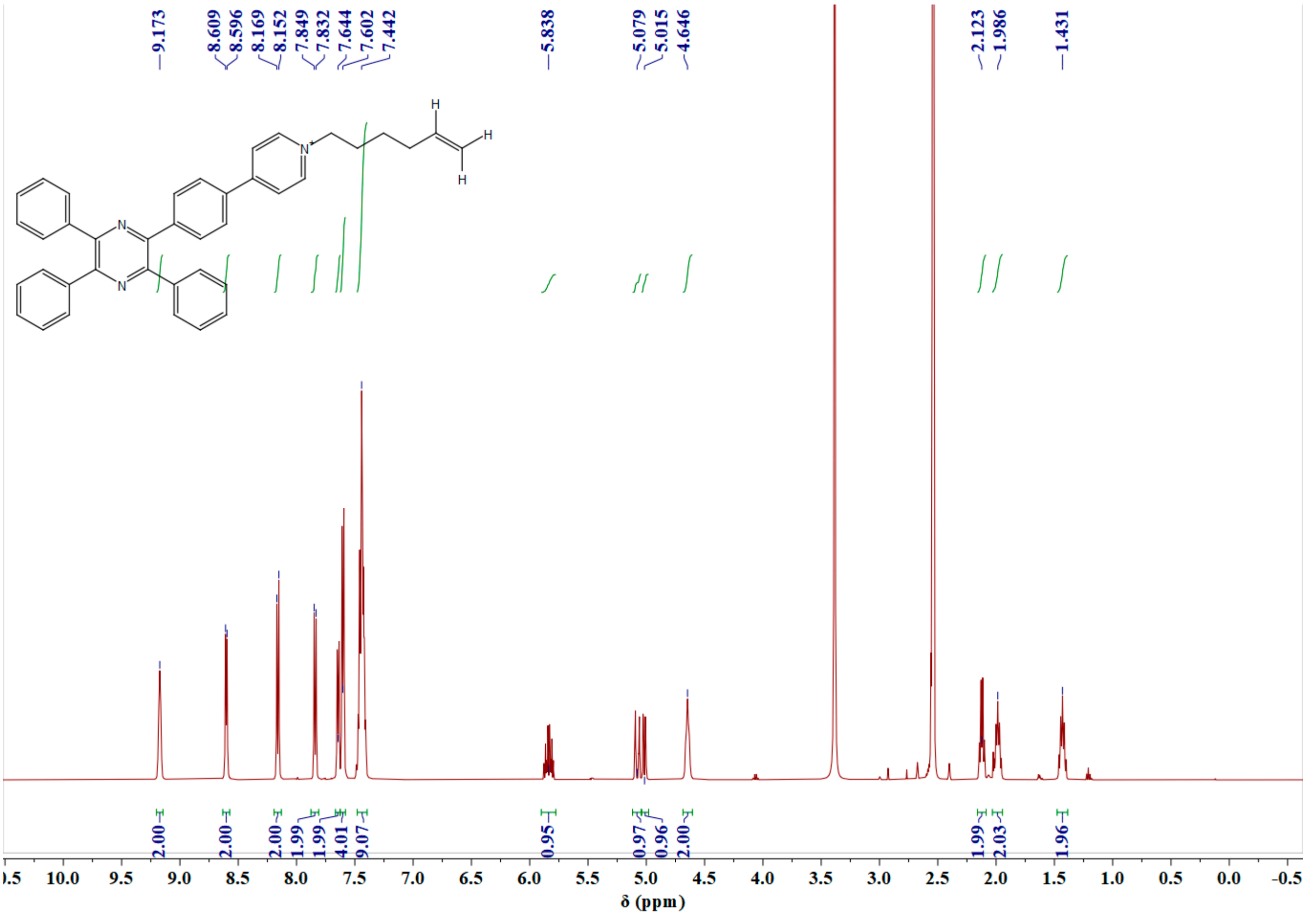

Figure S7. ^{1}H NMR of spectrum of compound **3** in DMSO-d_6.

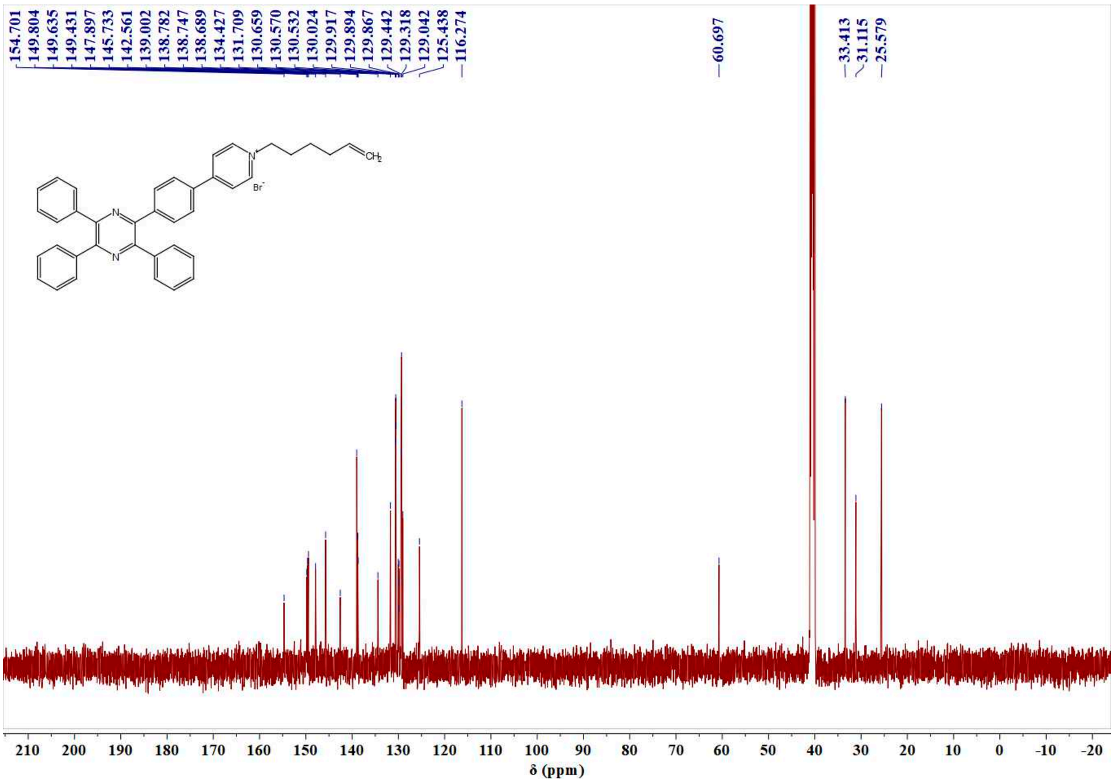

Figure S8. ^{13}C NMR of spectrum of compound **3** in $CDCl_3$.

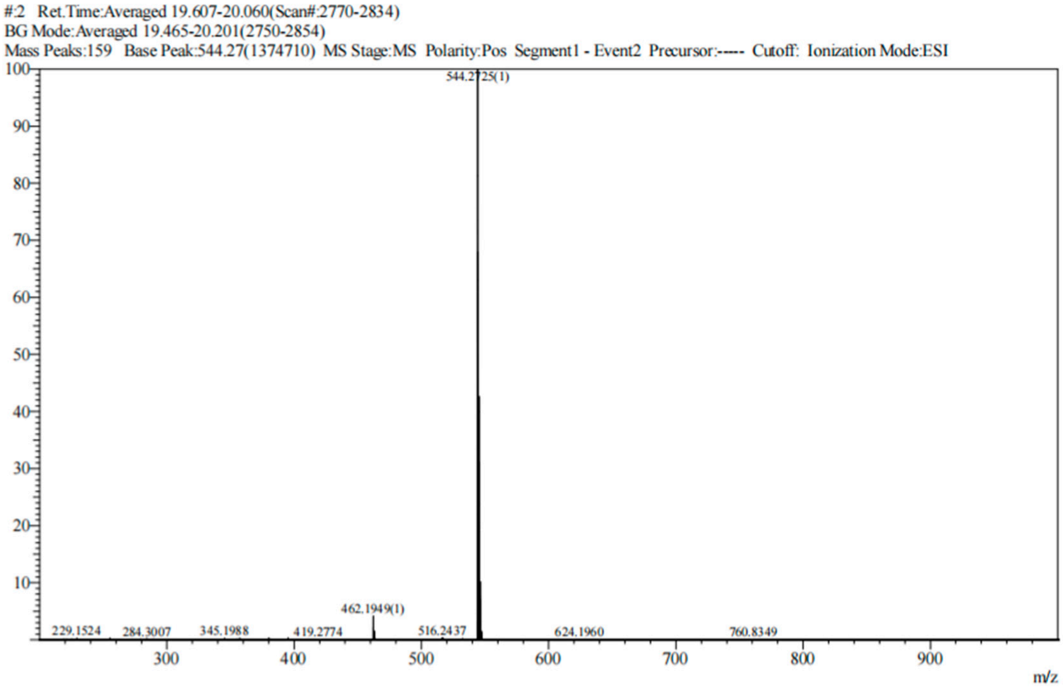

Figure S9. LCESI-MS spectrum of compound **3**.

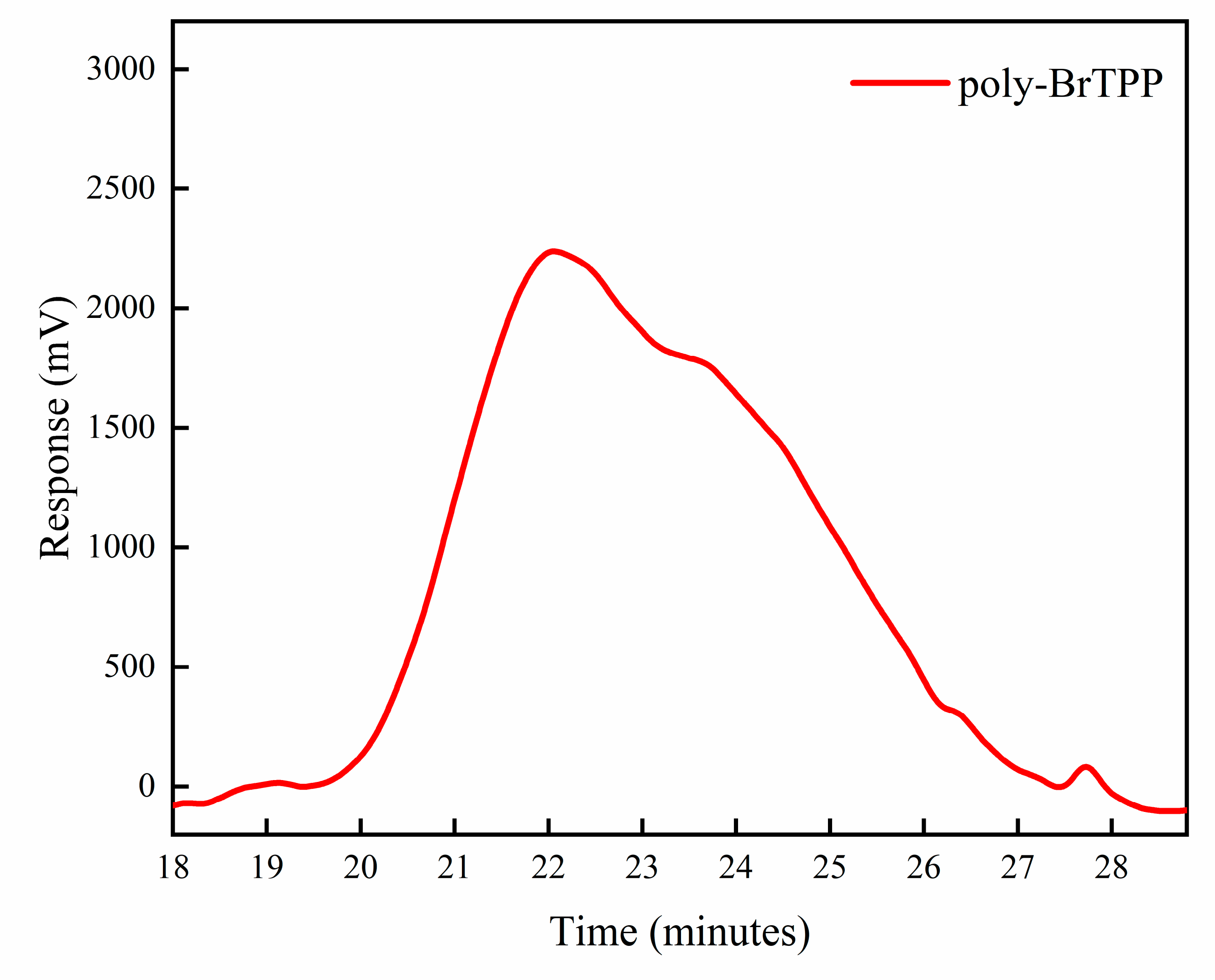

Figure S10. GPC chromatogram plot of poly-BrTPPz.

Table S1. GPC data of Poly-BrTPPz.

Name	Mp	Mn	Mw	Mv	Mz	Mz+1	PD
Poly-BrTPPz	13882	4026	10198	9043	19502	28552	2.533

Table S2. Photophysical properties and temperature-dependent emission of Br-TPPz and poly-Br-TPPz

Sample	λ_{abs} (nm)	λ_{em} (nm)	τ (µs)	ΔE_{ST} (eV)	PLQY (%)	λ_{em} (nm) at Different Temperatures (K)					
						78K	213K	233K	253K	273K	293K
Br-TPPz	350-360	500	-	0.3	-	–	–	–	–	–	–
Poly-BrTPPz	355	480	303	-	69.1	482	490	492	493	495	498

Computational Details and Optimized Coordinates

All density functional theory (DFT) and time-dependent DFT (TD-DFT) calculations were performed utilizing the **Gaussian 09** program package. Geometry optimizations for the ground state (S_0) and the lowest excited singlet/triplet states (S_1, T_1) of the monomer **Br-TPPz** were executed using the **M06-2X** global hybrid functional in combination with the **def2-TZVP** triple-zeta basis set. To realistic mimic the aqueous testing environment, the **SMD (Solvation Model based on Density)** implicit universal solvation model was applied with water as the solvent.

The default convergence criteria of the Berny optimization algorithm in Gaussian 09 were strictly enforced throughout the structural relaxing simulations:

Maximum Force: 0.000450 Hartree/Bohr

RMS Force: 0.000300 Hartree/Bohr

Maximum Displacement: 0.001800 Bohr

RMS Displacement: 0.001200 Bohr

The optimized Cartesian coordinates in the standard orientation (with the origin situated at the center of mass) are tabulated below:

Table S3. Optimized Cartesian coordinates (in AA) for the ground state (S_0) of Br-TPPz monomer in aqueous solution (M06-2X/def2-TZVP, SMD model).

Center	Atomic	Atomic	Coordinates (Angstroms)		
Number	Number	Type	X	Y	Z
1	6	0	4.090682	-0.986129	0.209382
2	6	0	4.793718	0.235759	-0.004366
3	7	0	4.124175	1.385696	0.082890
4	6	0	2.796436	1.404354	0.215449
5	6	0	2.083930	0.178255	0.103315
6	7	0	2.758048	-0.970887	0.180581
7	6	0	0.622325	0.075602	-0.119141
8	6	0	-0.060878	0.983265	-0.948120
9	6	0	-1.427079	0.854176	-1.165587

Table S3 (Continued)

Center Number	Atomic Number	Atomic Type	Coordinates (Angstroms) X	Y	Z
10	6	0	-2.160105	-0.189543	-0.565475
11	6	0	-1.467807	-1.108765	0.247411
12	6	0	-0.100258	-0.980142	0.462588
13	6	0	4.741015	-2.290857	0.483339
14	6	0	5.887366	-2.379682	1.292428
15	6	0	6.465646	-3.619925	1.564382
16	6	0	5.911443	-4.788129	1.029291
17	6	0	4.764729	-4.709720	0.231973
18	6	0	4.178587	-3.470850	-0.032542
19	6	0	6.234159	0.323974	-0.346513
20	6	0	6.840416	-0.613323	-1.201513
21	6	0	8.188453	-0.492487	-1.539963
22	6	0	8.951938	0.561681	-1.026245
23	6	0	8.354194	1.504543	-0.183215
24	6	0	7.002892	1.391717	0.147615
25	6	0	2.165564	2.716323	0.496345
26	6	0	2.683088	3.880027	-0.098010
27	6	0	2.111245	5.125052	0.167924
28	6	0	1.024751	5.226548	1.043495
29	6	0	0.517023	4.075656	1.656179
30	6	0	1.081648	2.829031	1.384814
31	6	0	-3.610727	-0.315446	-0.783452
32	6	0	-4.404307	0.795276	-1.140184
33	6	0	-5.761890	0.652582	-1.339335
34	7	0	-6.365926	-0.550006	-1.199239

Table S3 (Continued)

Center Number	Atomic Number	Atomic Type	Coordinates (Angstroms) X	Y	Z
35	6	0	-5.640254	-1.638401	-0.854341
36	6	0	-4.279030	-1.550189	-0.645995
37	6	0	-7.834383	-0.656819	-1.353860
38	6	0	-8.563353	-0.355293	-0.046826
39	6	0	-10.079053	-0.468746	-0.207105
40	6	0	-10.832453	-0.173220	1.097828
41	6	0	-12.323814	-0.245441	0.936606
42	6	0	-13.129895	-1.094406	1.581304
43	1	0	0.485135	1.788770	-1.439603
44	1	0	-1.920542	1.557581	-1.837143
45	1	0	-2.004757	-1.917404	0.744634
46	1	0	0.425206	-1.692702	1.098893
47	1	0	6.322039	-1.474408	1.718912
48	1	0	7.352127	-3.675601	2.200367
49	1	0	6.369998	-5.757623	1.237509
50	1	0	4.325246	-5.618350	-0.186280
51	1	0	3.281188	-3.401126	-0.649270
52	1	0	6.250783	-1.434005	-1.612560
53	1	0	8.645192	-1.223392	-2.211202
54	1	0	10.009217	0.650467	-1.286654
55	1	0	8.943927	2.331891	0.218467
56	1	0	6.527166	2.126035	0.799351
57	1	0	3.533522	3.791971	-0.775804
58	1	0	2.514365	6.020711	-0.310483
59	1	0	0.577771	6.201230	1.252543

Table S3 (Continued)

Center Number	Atomic Number	Atomic Type	Coordinates (Angstroms) X	Y	Z
60	1	0	-0.320604	4.150055	2.353433
61	1	0	0.686832	1.938123	1.875693
62	1	0	-3.973118	1.789305	-1.245565
63	1	0	-6.403479	1.491015	-1.609812
64	1	0	-6.184563	-2.578259	-0.763224
65	1	0	-3.739502	-2.462983	-0.399073
66	1	0	-8.129769	0.043547	-2.145992
67	1	0	-8.051659	-1.673313	-1.706327
68	1	0	-8.212831	-1.053337	0.731827
69	1	0	-8.295532	0.659715	0.291894
70	1	0	-10.419246	0.226831	-0.994353
71	1	0	-10.340653	-1.482527	-0.555969
72	1	0	-10.505759	-0.875782	1.883098
73	1	0	-10.551179	0.839670	1.441109
74	1	0	-12.759709	0.457375	0.213741
75	1	0	-14.210537	-1.100108	1.412179
76	1	0	-12.736100	-1.814841	2.306764
